# No *SMAD4* hypermethylation in colorectal cancer

**DOI:** 10.1054/bjoc.2000.1387

**Published:** 2000-10

**Authors:** S Roth, P Laiho, R Salovaara, V Launonen, L A Aaltonen

**Affiliations:** Department of Medical Genetics, Haartman Institute, University of Helsinki, Finland; Department of Pathology, Haartman Institute, University of Helsinki, Finland

**Keywords:** *SMAD4*, colorectal cancer, hypermethylation, promoter

## Abstract

The chromosome region 18q21 is frequently deleted in colorectal cancers. Three candidate tumour suppressor genes, *DCC*, *SMAD4* and *SMAD2*, map to this region. The *SMAD4(DPC4)* gene was recently identified as a candidate pancreatic cancer suppressor gene. It is also a gene for juvenile polyposis tumour predisposition syndrome. Somatic *SMAD4* mutations have been detected in some colorectal carcinomas. However, the frequency of these mutations is relatively low, and whether *SMAD4* plays a key role in colorectal tumorigenesis is still unclear. In addition to loss of chromosomal material and intragenic mutations there is a third mechanism, DNA methylation, which may have an important role in gene inactivation. In the present study, we examined whether promoter hypermethylation could be a mechanism for *SMAD4* inactivation. In total, 42 colorectal tumours were selected for the methylation analysis and no evidence of promoter hypermethylation was found. Our result suggests that hypermethylation of the *SMAD4* promoter region is not a frequent event in colorectal tumorigenesis. © 2000 Cancer Research Campaign


British Journal of Cancer (2000) 83(8), 1015–1019
© 2000 Cancer Research Campaign
doi: 10.1054/ bjoc.2000.1387, available online at http://www.idealibrary.com on IDEAL®

### No *SMAD4* hypermethylation in colorectal cancer

**S Roth,[1] P Laiho,[1] R Salovaara,[1,2] V Launonen[1] and LA Aaltonen[1]**

[1]Department of Medical Genetics, Haartman Institute, University of Helsinki, Finland; [2]Department of Pathology, Haartman Institute, University of Helsinki, Finland

**Summary** The chromosome region 18q21 is frequently deleted in colorectal cancers. Three candidate tumour suppressor genes, *DCC*, *SMAD4* and *SMAD2*, map to this region. The *SMAD4(DPC4)* gene was recently identified as a candidate pancreatic cancer suppressor gene. It is also a gene for juvenile polyposis tumour predisposition syndrome. Somatic *SMAD4* mutations have been detected in some colorectal carcinomas. However, the frequency of these mutations is relatively low, and whether *SMAD4* plays a key role in colorectal tumorigenesis is still unclear. In addition to loss of chromosomal material and intragenic mutations there is a third mechanism, DNA methylation, which may have an important role in gene inactivation. In the present study, we examined whether promoter hypermethylation could be a mechanism for *SMAD4* inactivation. In total, 42 colorectal tumours were selected for the methylation analysis and no evidence of promoter hypermethylation was found. Our result suggests that hypermethylation of the *SMAD4* promoter region is not a frequent event in colorectal tumorigenesis. © 2000 Cancer Research Campaign

**Keywords:** *SMAD4*; colorectal cancer; hypermethylation; promoter

Colorectal carcinogenesis is a multistep process, and tumour progression is promoted by a series of genetic changes that involve activation of oncogenes and inactivation of tumour suppressor genes (Vogelstein et al, 1988; Kinzler and Vogelstein, 1998). The prominent role of some tumour suppressor genes such as *APC* (5q) and *p53* (17p) has been widely recognized. Several other tumour suppressor genes have been suggested to exist e.g. on chromosome bands 1p, 8p, 18q and 22q, and inactivation of these genes may play a role in colorectal carcinogenesis (Vogelstein et al, 1988; Miyaki et al, 1999).

Previous studies have demonstrated that at least one copy of chromosome 18q is lost in over 70 percent of sporadic colorectal cancers (Vogelstein et al, 1988; Ried et al, 1996; Meijer et al, 1998; Korn et al, 1999). Three candidate tumour suppressor genes, *DCC*, *SMAD4* and *SMAD2*, map to this region. Loss of expression of *DCC* has been reported in advanced colorectal carcinomas, but mutations in the coding region seem to be rare, and the position of *DCC* as a candidate tumour suppressor is still not clear (Fearon et al, 1990; Kikuchi-Yanoshita et al, 1992; Cho et al, 1994). *SMAD4*, originally named as *DPC4*, is frequently deleted in pancreatic cancers (Hahn et al, 1996). Loss of the *SMAD4* region relatively rarely occurs in other types of tumours. An important exception is colorectal cancer, in which there is good evidence for allelic loss at this locus (Takagi et al, 1996; Thiagalingham et al, 1996). Mutations of *SMAD4* and *SMAD2* genes have been detected in some colorectal carcinomas, but the frequency of these mutations is relatively low (Eppert et al, 1996; Takagi et al, 1996; Thiagalingham et al, 1996; MacGrogan et al, 1997).

Knudson's hypothesis that two hits are required for the full inactivation of a tumour suppressor gene has been shown to be correct in many human cancers (Jones and Laird, 1999). Traditionally, it has been thought that intragenic mutations and loss of chromosomal material inactivate tumour suppressor genes. However, the fact that methylation of CpG islands located in the promoters of genes can cause transcriptional silencing, has led to the suggestion that hypermethylation of tumour suppressor gene promoters is one of the mechanisms promoting malignant transformation (Jones and Laird, 1999). The pattern characterized by methylation in tumour tissue and lack of methylation in normal mucosa is consistent with the CpG island methylator phenotype (CIMP), typical for the colorectal cancers displaying microsatellite instability (MSI) (Toyota et al, 1999). On the other hand, the promoter hypermethylation in normal mucosa and lack of methylation in tumour tissue have been reported in sporadic microsatellite stable (MSS) colorectal cancers (Kuismanen et al, 1999). Recently, the involvement of SMAD4 in sporadic colorectal neoplasia was studied by immunohistochemistry (Salovaara et al, submitted) and it was shown that SMAD4 expression was considerably reduced in unselected colorectal carcinomas. This prompted us to examine whether promoter hypermethylation plays a role in *SMAD4* inactivation.

One candidate region for the *SMAD4* promoter was first reported by Minami et al (1998). They cloned a 1.4-kb fragment of the *SMAD4* 5′-flanking region from phage library by using the first coding exon's sequence as a primer. This *SMAD4* promoter lacks typical TATA boxes and CpG island, but contains some TATA-like structures (TAAAAT) as well as some binding sites for transcription factors (Minami et al, 1998). Another candidate sequence for the *SMAD4* promoter has been characterized by Hagiwara et al (submitted). This region locates upstream from the previously reported coding exons and it includes a new non-coding exon (exon 1), CpG island as well as TATAA and CCAAT boxes and consensus SP1 binding site (GGGCGGG).

The aim of this study was to confirm the low frequency of *SMAD4* mutations in colorectal cancer and to evaluate the role of promoter region alterations in *SMAD4* inactivation.

*Received 25 January 2000*
*Revised 20 April 2000*
*Accepted 15 June 2000*

*Correspondence to:* LA Aaltonen

**Table 1** List of samples included into *SMAD4* methylation analysis. Tumour stages are shown as Dukes' stages (A = tumour limited to mucosa and submucosa; B = tumour penetrating the muscle wall; C = metastases to regional lymph nodes; D = distant metastases). The proportion of tumour tissue is shown as percentage. Among 42 sample pairs, 26 tumours were previously shown to be MSI (Aaltonen et al, 1998[1]; Salovaara et al, 2000). Either normal mucosa (NM) or blood (B) was used as a source of normal tissue for DNA extraction. The presence of SMAD4 protein has been previously analysed from 18 out of 42 tumour samples (Salovaara et al, submitted), and 5 of them were SMAD4 negative. An asterisk marks the 9 samples also included into mutation analysis.

| Sample No. | Dukes' stage | Tumour % | MSS/ (ref) MSI | Origin of normal DNA | Immunostaining by Salovaara et al |
|---|---|---|---|---|---|
| C104* | B | 80 | MSI[1] | NM | Not included |
| C144* | B | 90 | MSI[1] | NM | Not included |
| C145* | C | 70 | MSI[1] | NM | Ca+ |
| C171* | B | 75 | MSI[1] | B | Ca+ |
| C287 | A | 75 | MSI[1] | B | Ca+ |
| C406* | C | 85 | MSI[1] | NM | Not included |
| C484 | D | 80 | MSI[1] | NM | Ca+ |
| C500 | B | 75 | MSI[1] | NM | Ca+ |
| C521 | D | 70 | MSI[1] | NM | Ca+ |
| C526 | C | 90 | MSS[1] | NM | Not included |
| C532 | B | 75 | MSI[1] | NM | Ca+ |
| C543 | ? | 90 | MSI[1] | NM | Not included |
| C549* | A | 80 | MSI[1] | NM | Not included |
| C567 | B | 85 | MSI[1] | NM | Not included |
| C568 | B | 70 | MSI[1] | NM | Ca+ |
| C578 | B | 85 | MSI[1] | NM | Ca+ |
| C732 | A | 60 | MSI[2] | NM | Not included |
| C733 | C | 90 | MSS[2] | NM | Not included |
| C744 | B | 70 | MSI[2] | B | Ca+ |
| C758 | B | 85 | MSI[2] | B | Not included |
| C768 | C | 55 | MSI[2] | NM | Not included |
| C777 | B | 65 | MSI[2] | NM | Not included |
| C778 | C | 90 | MSI[2] | NM | Not included |
| C789 | A | 50 | MSI[2] | NM | Not included |
| C800 | B | 70 | MSI[2] | NM | Not included |
| C813 | B | 90 | MSS[2] | NM | Not included |
| C844 | B | 70 | MSI[2] | NM | Not included |
| C846 | B | 90 | MSS[2] | NM | Not included |
| C883 | B | 85 | MSI[2] | B | Ca+ |
| C941 | B | 75 | MSI[2] | NM | Not included |
| C961 | B | 95 | MSS[2] | B | Ca– |
| C962 | C | 70 | MSS[2] | B | Not included |
| C964 | B | 75 | MSS[2] | B | Not included |
| C978* | C | 60 | MSS[2] | B | Ca– |
| C982 | C | 60 | MSS[2] | B | Ca– |
| C984* | B | 60 | MSS[2] | B | Ca+ |
| C986 | C | 55 | MSS[2] | B | Ca– |
| C988 | C | 50 | MSS[2] | B | Ca– |
| C989 | C | 65 | MSS[2] | B | Ca+ |
| C1036 | C | 90 | MSS[2] | NM | Not included |
| C1051* | D | 55 | MSS[2] | B | Not included |
| C1058 | B | 85 | MSS[2] | NM | Not included |

### MATERIALS AND METHODS

### Patients and tissue preparations

Over one thousand fresh-frozen colorectal adenocarcinoma specimens were collected in the Department of Medical Genetics, Haartaman Institute, University of Helsinki between May 1994 and June 1998 (Aaltonen et al, 1998; Salovaara et al, 2000). To document the proportion of tumour tissue, all specimens had been examined histologically. Either normal mucosa or blood was used as a source of normal tissue for DNA extraction. The MSI status of the tumours had been determined previously (Aaltonen et al, 1998; Salovaara et al, 2000). Samples included in the present study are listed in Tables 1 and 2. The proportion of tumour tissue, stage of cancers (as Dukes' stages), MSI status and source of normal tissue DNA are also presented. 24 out of 55 colorectal tumours included into this study have been previously analysed by immunohistochemistry for the presence of SMAD4 protein and the SMAD4 expression was below detection level in seven of them (Salovaara et al, submitted). The antibody used for immunostaining was monoclonal antibody to SMAD4 (B-8, Santa Cruz-7966), which has been previously reported to function well also on paraffin embedded tissue sections (Wilentz et al, 2000).

### *SMAD4* PROMOTER METHYLATION

The methylation status of the *SMAD4* promoter was studied in 26 MSI and 16 MSS colorectal cancers (CRC). The fragment selected for this analysis was a CG-rich region characterized by Hagiwara

 © 2000 Cancer Research Campaign

**Table 2** Tumour samples included into *SMAD4* mutation screening. Tumour stages are shown as Dukes' stages. The proportion of tumour tissue is presented as percentage. Among 22 tumours, 15 were MSI, seven being MSS. The MSI/MSS status has been reported previously by Aaltonen et al (1998)[1] and Salovaara et al (2000)[2]. 10 out of 22 tumours have been previously analysed by SMAD immunostaining (Salovaara et al, submitted)

| Sample No. | Dukes' stage | Tumour % | MSS/ (ref) MSI | Immunostaining by Salovaara et al |
|---|---|---|---|---|
| C11 | B | 80 | MSI[1] | Ca– |
| C18 | A | 90 | MSI[1] | Ca+ |
| C43 | A | 70 | MSI[1] | Not included |
| C54 | D | 60 | MSI[1] | Not included |
| C64 | A | 70 | MSI[1] | Ca+ |
| C104 | B | 80 | MSI[1] | Not included |
| C136 | B | 75 | MSI[1] | Ca+ |
| C144 | B | 90 | MSI[1] | Ca+ |
| C145 | C | 70 | MSI[1] | Ca+ |
| C171 | B | 75 | MSI[1] | Ca+ |
| C204 | B | 70 | MSI[1] | Not included |
| C239 | B | 90 | MSI[1] | Not included |
| C331 | B | 90 | MSI[1] | Not included |
| C406 | C | 85 | MSI[1] | Not included |
| C549 | A | 80 | MSI[1] | Not included |
| C977 | B | 65 | MSS[2] | Ca– |
| C978 | C | 60 | MSS[2] | Ca– |
| C983 | D | 70 | MSS[2] | Not included |
| C984 | B | 60 | MSS[2] | Ca+ |
| C1051 | D | 55 | MSS[2] | Not included |
| C1083 | D | 75 | MSS[2] | Not included |
| C1088 | B | 75 | MSS[2] | Not included |

et al (submitted), including the non-coding exon 1 (Figure 1). To determine whether this region was hypermethylated, a PCR-based *HpaII* and *MspI* restriction enzyme assay was used. This assay is based on the ability of the *HpaII* restriction enzyme to distinguish CpG sites that are methylated versus those that are nonmethylated. If the restriction sites are methylated, the methylation-sensitive *HpaII* can not cleave the DNA while *MspI*, which is the methylation-insensitive isoschizomer of *HpaII*, is capable to cleave. After digestion with these enzymes, *SMAD4* primers flanking the *HpaII/MspI* site are used to test the *HpaII* and *MspI* treated DNA for PCR amplification. PCR product should be detected only when the original target DNA contains methylated *HpaII* restriction sites.

Both tumour and normal DNA were digested and the reactions contained either no enzyme, 25 units of *HpaII*, or 20 units of *MspI* for 16 h at 37°C. To analyse cleavage of the *SMAD4* promoter region, 12.5 ng of DNA from each digest was analysed by PCR in 25 µl reactions containing 1× PCR reaction buffer (PE/ABI), 100 µM of each dNTP (Finnzymes), 1.5 mM of $MgCl_2$, 1 unit of AmpliTaqGOLD polymerase (PE/ABI), 10% of DMSO and 0.4 µM of each primer. Primers were designed (Primer3) to amplify 408 bp fragment of the *SMAD4* promoter (Hagiwara et al, submitted) containing six *HpaII*/*MspI* restriction sites (Figure 1) and the primer sequences were: forward: 5′-CAAGTTGGCAGCAA-CAACAC; and reverse: 5′-ACATGGCGCGGTTACCT. PCR was performed for one cycle of 95°C for 10 min followed by 40 cycles of 95°C for 30 s, 60°C for 45 s, and 72°C for 45 s, followed by one cycle of 72°C for 10 min. The resulting PCR products were analysed by agarose gel electrophoresis (3% agarose gel).

**Figure 1** *SMAD4* promoter region studied for methylation. The primers designed for this analysis are underlined. ccgg indicates all *HpaII/MspI* restriction sites in this region. Grey shading depicts the noncoding exon 1

methylation analysis. These tumours were unselected regarding Dukes' stages A and B (4 tumours of stage A, 11 of stage B, 3 of stage C, and 4 of stage D, see Table 2). *SMAD4* was amplified from genomic DNA by using previously published primers (Roth et al, 1999; Zhou et al, 1999). The PCR-reactions were carried out in 50 µl reaction volume including 100 ng genomic DNA, 1 × PCR reaction buffer (Perkin Elmer Applied Biosystems Division), 200 µM of each dNTP (Finnzymes), 0.8 µM of each primer, and 2 units of AmpliTaqGOLD polymerase (PE/ABI). The $MgCl_2$ concentration was 1.5 mM in all reactions except for untranslated fragment, where the magnesium concentration was 2.5 mM. The following PCR cycles were used for amplification: exons 1, 2, and 11 – 10 min at 95°C, 40 cycles of 45 s at 95°C, 45 s at 57°C, 1 min at 72°C; for exons 3,5, and 6 – 10 min at 95°C, 40 cycles of 45 s at 95°C, 45 s at 58°C, 1 min at 72°C; for exons 4, 7, 8, 9, and 10 – 10 min at 95°C, 40 cycles of 45 s at 95°C, 45 s at 56°C, 1 min at 72°C; for 5′-untranslated fragment – 10 min at 95°C, 40 cycles of 1 min at 95°C, 1 min at 56°C, 1 min 30 s at 72°C. Final extension 10 min at 72°C was used for all fragments. After PCR, 5 µl of the

### Mutation analysis of *SMAD4*

*SMAD4* mutations were analysed among 15 MSI and seven MSS tumours from patients with CRC, nine of those being same as in

© 2000 Cancer Research Campaign 

PCR product was run in a 3% agarose (NuSieve) gel to verify the specificity of the PCR reaction. The rest of the PCR product was purified using QIAquick PCR purification kit (QIAGEN). Direct sequencing of the PCR products was performed using the ABI PRISM Dye Terminator or ABI PRISM dRhodamine cycle sequencing kits (PE/ABI). Cycle sequencing products were electrophoresed on 6% Long Ranger gels (FMC Bioproducts) and analysed on an Applied Biosystems model 373A or 377 DNA sequencer (PE/ABI).

### Restriction enzyme digestion

To screen for the presence of a base substitution in exon 2 in control individuals, restriction enzyme digestion was performed. *NsiI* (New England BioLabs) digestion was used to detect the G to A change at codon 118. *NsiI* cuts the PCR fragment (530 bp), which contains the base substitution into two fragments (264 bp and 266 bp by size) whereas the wild-type fragment lacks the restriction site and is not digested. The PCR was performed as described above (*SMAD4* mutation analysis, exon 2). The digestion was performed in 1 × NEBuffer (New England BioLabs) at 37°C overnight. After digestion, the PCR products were electrophoresed through 3% agarose gel.

### RESULTS

### *SMAD4* promoter methylation

In this study, the methylation status of the *SMAD4* promoter was analysed using *HpaII* and *MspI* digestion. Using this assay, we examined the methylation status for *SMAD4* promoter region in a group of 26 MSI and 16 MSS colorectal tumours (Table 1). The amplified sequence contained altogether 55 CpG dinucleotides of which the methylation status for six CCGG sites was possible to determine by restriction (Figure 1). PCR amplification was not detected from any of *HpaII* digested DNA, suggesting that the *SMAD4* promoter is unmethylated in all cases studied. The non-digested tumour and normal DNA showed amplification in all samples, demonstrating the efficiency of PCR reaction (Figure 2).

### *SMAD4* mutation analysis

Twenty-two primary colon cancers were analysed for mutations of all exons of *SMAD4* by genomic sequencing (Table 2). Also part of the *SMAD4* 5′-untranslated region (331 bp fragment downstream from the transcription start site) published by Minami et al (1998) was included in mutation screening. The only change detected was G to A transition at the third position for codon 118 (exon 2). This silent change was present in one tumour sample (C11) and also in corresponding normal DNA. We analysed the frequency of this variant among 84 Finnish cancer free control individuals by restriction enzyme digestion (*NsiI*), and the change was found in one.

### DISCUSSION

Loss of heterozygosity (LOH) on chromosome 18 q is frequently detected during the progression of colorectal carcinomas. This suggests the presence of a tumour suppressor gene or genes at this region. Since identification of *SMAD4*/*DPC4*, mutation analyses of this gene have been carried out in many cancer types. However,

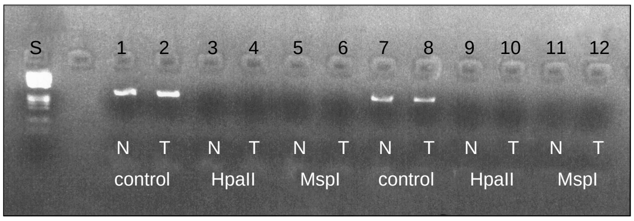

**Figure 2** Methylation analysis of CpG islands of *SMAD4* promoter in two colorectal cancer samples (C789, lanes 1–6 and C800, lanes 7–12). Lanes 1, 2, 7 and 8: PCR product from nondigested normal (N) and tumour (T) DNA. Lanes 3, 4, 9 and 10: lack of PCR product from *HpaII* digested normal and tumour DNA. Lanes 5, 6, 11 and 12: lack of PCR product from *MspI* digested normal and tumour DNA

a relatively low frequency of mutations (<10%) has been found in most cancer types; the only exception being pancreatic cancer, where the mutation frequency is approximately 20% (Hahn et al, 1996; Nagatake et al, 1996; Scutte et al, 1996; Takagi et al, 1996; Thiagalingham et al, 1996; Rozenblum et al, 1997).

In addition to loss of chromosomal material and intragenic mutations there is a third mechanism, DNA methylation, which may have important role in inactivation of tumour suppressor genes. Two types of DNA methylation changes appear to be connected with the progression of malignant tumours; hypomethylation induced activation of oncogenes and hypermethylation based silencing of tumour suppressor genes (Laird and Jaenish, 1994). Main targets of hypermethylation are normally unmethylated CpG islands locating in gene promoter regions. Methylation of cytosine at CpG dinucleotides of 5′ CpG islands has been associated with transcriptional silencing of tumour suppressor genes in a variety of human cancers (Jones and Laird, 1999). For example, *VHL* gene promoter is commonly hypermethylated in renal cancers and *RBI* gene in retinoblastoma, respectively (Sakai et al, 1991; Herman et al, 1994).

In the present study, we examined whether hypermethylation of promoter could be an alternative mechanism to coding region mutations for *SMAD4* inactivation. The CpG island near noncoding exon 1 was selected for this analysis (Figure 1), since it is well documented that methylation has important regulatory effects especially when involving these CpG rich areas (Bird, 1986). Twenty-six MSI and 16 MSS tumour and corresponding normal DNAs were selected for the analysis and no evidence of hypermethylation was found. The region analysed here contains many promotor associated structures. While we cannot exclude the existence of other relevant sequences, we consider the region analysed here as a likely candidate for the *SMAD4* promoter. Six CCGG sites were available for methylation analysis by restriction. The possibility, that methylation of other CpG sites are important in silencing the *SMAD4* gene cannot be excluded. Bearing these cautions in mind, our data suggests that the hypermethylation of the *SMAD4* promoter region is not a key mechanism in SMAD4 inactivation.

In a recent study by Zhou et al (1999) two mutations were identified in the *SMAD4* 5′-untranslated region among the 6 endometrial tumours that had previously failed to express wild type *SMAD4* (Zhou et al, 1999). In that study the mutation screening was focused on a 331 bp long fragment, which spanned nucleotides –262 to +69 from the transcription start site. This fragment is part of the *SMAD4* 5′-untranslated region published by Minami et al (1998) and it contains several important transcription factor binding sites (Minami et al, 1998). To examine whether the region is mutated also in colorectal carcinomas, we sequenced this

 *© 2000 Cancer Research Campaign*

331-bp fragment from 15 MSS and seven MSI tumour samples and found no changes.

Miyaki et al (1999) analysed *SMAD4* mutations from 176 colorectal tumours and found that *SMAD4* mutation frequencies were 0% in adenoma, 10% in intramucosal carcinoma, 7% in primary invasive carcinoma without metastasis, 35% in primary invasive carcinoma with metastasis and 31% in distant metastasis. Thus, the frequency of *SMAD4* mutations was correlating with the stage of colorectal cancer (Miyaki et al, 1999). Similar results were also published by Koyama et al (1999). They found somatic *SMAD4* mutation in 7 of 64 (11%) colorectal tumours of clinical stages II or III, all these tumours also showing LOH at 18q21. In the present study, 22 primary CRCs were selected into the *SMAD4* mutation screening and the only change detected was a polymorphism in exon 2 (C11). According to the literature, *SMAD4* polymorphisms are rare, so the frequency of this variant among Finnish control individuals was evaluated. In total, 84 controls were analysed and the change was found in one of them.

Our results suggest that *SMAD4* is not frequently mutated in primary non-metastatic colorectal carcinoma and that the hypermethylation of the *SMAD4* promoter region is not a key mechanism in colorectal tumorigenesis. As expression of SMAD4 appears to be decreased or lost in most colorectal cancers (Salovaara et al, submitted), further work is necessary to understand the molecular mechanisms of *SMAD4* inactivation.

### ACKNOWLEDGEMENTS

We would like to thank Emil Aaltonen Foundation, Sigrid Juselius Foundation, Finnish Cancer Society, the Academy of Finland, Finnish Cultural Foundation and Biocentrum Helsinki for supporting this work and Siv Lindroos for technical assistance.

### REFERENCES

Aaltonen LA, Salovaara R, Kristo P, Canzian F, Hemminki A, Peltomäki P, Chadwick R, Kääriäinen H, Eskelinen M, Järvinen H, Mecklin J-P and de la Chapelle A (1998) Incidence of hereditary nonpolyposis colorectal cancer and the feasibility of molecular screening for the disease. *N Eng J Med* **338**: 1481–1487

Bird AP (1986) CpG-rich islands and the function of DNA methylation. *Nature* **321**: 209–213

Cho KR, Oliner JD, Simons JW, Hedrick L, Fearon ER, Preisinger AC, Hedge P, Silverman GA and Vogelstein B (1994) The DCC gene: structural analysis and mutations in colorectal carcinomas. *Genomics* **19**: 525–531

Eppert K, Scherer SW, Ozcelik H, Pirone R, Hoodless P, Kim H, Tsui LC, Bapat B, Gallinger S, Andrulis IL, Thomsen GH, Wrana JL and Attisano L (1996) MADR2 maps to 18q21 and encodes a TGFbeta-regulated MAD-related protein that is functionally mutated in colorectal carcinoma. *Cell* **86**: 543–552

Fearon ER, Cho KR, Nigro JM, Kern SE, Simons JW, Ruppert JM, Hamilton SR, Preisinger AC, Thomas G, Kinzler KW and Vogelstein B (1990) Identification of a chromosome 18q gene that is altered in colorectal cancers. *Science* **247**: 49–56

Hahn SA, Schutte M, Hoque AT, Moskaluk CA, da Costa LT, Rozenblum E, Weinstein CL, Fischer A, Yeo CJ, Hruban RH and Kern SE (1996) DPC4, a candidate tumor suppressor gene at human chromosome 18q21.1. *Science* **271**: 350–353

Herman JG, Latif F, Weng Y, Lerman MI, Zbar B, Liu S, Samid D, Duan DS, Gnarra JR and Linehan WM (1994) Silencing of the VHL tumor suppressor gene by DNA methylation in renal carcinoma. *Proc Natl Acac Sci USA* **91**: 9700–9704

Jones PA and Laird PW (1999) Cancer epigenetics comes of age. *Nat Genet* **21**: 163–167

Kikuchi-Yanoshita R, Konishi M, Fukunari H, Tanaka K and Miyaki M (1992) Loss of expression of the DCC gene during progression of colorectal carcinomas in familial adenomatous polyposis and non-familial adenomatous polyposis patients. *Cancer Res* **52**: 3801–3803

Kinzler KW and Vogelstein B (1998) Colorectal tumours. In: *The Genetic Basis of Human Cancer*, Vogelstein B and Kinzler KW (eds) pp. 565–587. McGraw-Hill Press: United States

Korn WM, Yasutake T, Kuo WL, Warren RS, Collins C, Tomita M, Gray J and Waldman FM (1999) Chromosome arm 20q gains and other genomic alterations in colorectal cancer metastatic to liver, as analysed by comparative genomic hybridisation and fluorescence in situ hybridisation. *Genes Chromosomes & Cancer* **25**: 82–90

Koyama M, Ito M, Nagai H, Emi M and Moriyama Y (1999) Inactivation of both alleles of the DPC4/SMAD4 gene in advanced colorectal cancers: identification of seven novel somatic mutations in tumours from Japanese patients. *Mutat Res* **406**: 71–77

Kuismanen SA, Holmberg MT, Salovaara R, Schweizer P, Aaltonen LA, de la Chapelle A, Nyström-Lahti M and Peltomäki P (1999) Epigenetic phenotypes distinguish microsatellite stable and -unstable colorectal cancers. *Proc Natl Acac Sci USA* **96**: 12661–12666

Laird PW and Jaenisch R (1994) DNA methylation and cancer. *Hum Molec Genet* **3**: 1487–1495

MacGrogan D, Pegram M, Slamon D and Bookstein R (1997) Comparative mutational analysis of DPC4 (SMAD4) in prostatic and colorectal carcinomas. *Ongogene* **15**: 1111–1114

Meijer GA, Hermsen MA, Baak JP, van Diest PJ, Meuwissen SG, Belien JA, Hoovers JM, Joenje H, Snijders PJ and Walboomers JM (1998) Progression from colorectal adenoma to carcinoma is associated with non-random chromosomal gains as detected by comparative genomic hybridisation. *J Clin Pathol* **51**: 901–909

Minami R, Kitazawa R, Maeda S and Kitazawa S (1998) Analysis of 5′-flanking region of human Smad4 (DPC4) gene. *Biochimica et Biophysica Acta* **1443**: 182–185

Miyaki M, Iijima T, Konishi M, Sakai K, Ishii A, Yasuno M, Hishima T, Koike M, Shitara N, Iwama T, Utsunomiya J, Kuroki T and Mori T (1999) Higher frequency of Smad4 gene mutation in human colorectal cancer with distant metastasis. *Oncogene* **20**: 3098–3103

Nagatake M, Takagi Y, Osada H, Uchida K, Mitsudomi T, Saji S, Shimokata K and Takahashi T (1996) Somatic in vivo alterations of the DPC4 gene at 18q21 in human lung cancers. *Cancer Res* **56**: 2718–2720

Ried T, Knutzen R, Steinbeck R, Blegen H, Schrock E, Heselmeyer K, du Manoir S and Auer G (1996) Comparative genomic hybridisation reveals a specific pattern of chromosomal gains and losses during the genesis of colorectal tumors. *Genes Chromosomes Cancer* **15**: 234–245

Roth S, Sistonen P, Hemminki A, Salovaara R, Loukola A, Avizienyte E, Lynch P, Amos C, Mecklin J-P, Kellokumpu I, Järvinen H and Aaltonen LA (1999) *SMAD* genes in juvenile polyposis. *Genes Chromosomes Cancer* **26**: 54–61

Rozenblum E, Schutte M, Goggins M, Hahn SA, Panzer S, Zahurak M, Goodman SN, Sohn TA, Hruban RH, Yeo CJ and Kern SE (1997) Tumour suppressive pathways in pancreatic carcinoma. *Cancer Res* **57**: 1731–1734

Sakai T, Toguchida J, Ohtani N, Yandell DW, Rapaport JM and Dryja TP (1991) Allele-specific hypermethylation of the retinoblastoma tumour suppressor gene. *Am J Hum Genet* **48**: 880–888

Salovaara R, Loukola A, Kristo P, Kääriäinen H, Ahtola H, Eskelinen M, Härkönen N, Julkunen R, Kangas E, Ojala S, Tulikoura J, Valkamo E, Järvinen H, Mecklin J-P, Aaltonen LA and de la Chapelle A (2000) Population-wide molecular detection of hereditary nonpolyposis colorectal cancer. *J Clin Oncol* **18**: 2193–2200

Schutte M, Hruban RH, Hedrick L, Cho KR, Nadasdy GM, Weinstein CL, Bova GS, Isaacs WB, Cairns P, Nawroz H, Sidransky D Jr, Casero RA, Meltzer PS, Hahn SA and Kern SE (1996) DPC4 gene in various tumour types. *Cancer Res* **56**: 2527–2530

Takagi Y, Kohmura H, Futamura M, Kida H, Tanemura H, Shimokawa K and Saji S (1996) Somatic alterations of the DPC4 gene in human colorectal cancers in vivo. *Gastroenterology* **111**: 1369–1372

Thiagalingam S, Lengauer C, Leach FS, Schutte M, Hahn SA, Overhauser J, Willson JK, Markowitz S, Hamilton SR, Kern SE, Kinzler KW and Vogelstein B (1996) Evaluation of candidate tumour suppressor genes on chromosome 18 in colorectal cancers. *Nat Genet* **13**: 343–346

Toyota M, Ahuja N, Ohe-Toyota M, Herman JG, Baylin SB and Issa J-P (1999) CpG island metylator phenotype in colorectal cancer. *Proc Natl Acac Sci USA* **96**: 8681–8686

Vogelstein B, Fearon ER, Hamilton SR, Kern SE, Preisinger AC, Leppert M, Nakamura Y, White R, Smits AM and Bos JL (1988) Genetic alterations during colorectal tumour development. *N Engl J Med* **319**: 525–532

Wilentz RE, Su GH, Dai JL, Sparks AB, Argani P, Sohn TA, Yeo CJ, Kern SE and Hruban RH (2000) Immunohistochemical labeling for Dpc4 Mirrors genetic status in pancreatic adenocarcinoma. *Am J Pathol* **156**: 37–43

Zhou Y, Kato H, Shan D, Minami R, Kitazawa S, Matsuda T, Arima T, Barrett JC and Wake N (1999) Involvement of mutations in the DPC4 promoter in endometrial carcinoma development. *Mol Carcinog* **25**: 64–72

*© 2000 Cancer Research Campaign*
